# Chitosan/Alginate Polymeric Nanoparticle-Loaded α-Mangostin: Characterization, Cytotoxicity, and In Vivo Evaluation against Breast Cancer Cells

**DOI:** 10.3390/polym15183658

**Published:** 2023-09-05

**Authors:** Muchtaridi Muchtaridi, Ade Irma Suryani, Nasrul Wathoni, Yedi Herdiana, Ahmed Fouad Abdelwahab Mohammed, Amirah Mohd Gazzali, Ronny Lesmana, I. Made Joni

**Affiliations:** 1 Department of Pharmaceutical Analysis and Medicinal Chemistry, Faculty of Pharmacy, Universitas Padjadjaran, Sumedang 45363, Indonesia; ade20011@mail.unpad.ac.id; 2 Functional Nano Powder University Center of Excellence (FiNder U CoE), Universitas Padjadjaran, Jalan Raya Bandung-Sumedang Km 21, Jatinangor 45363, Indonesia; 3 Research Collaboration Centre for Radiopharmaceuticals Theranostic, National Research and Innovation Agency (BRIN), Jakarta 10340, Indonesia; 4 Department of Pharmaceutics and Pharmaceutical Technology, Faculty of Pharmacy, Universitas Padjadjaran, Sumedang 45363, Indonesia; nasrul@unpad.ac.id (N.W.); y.herdiana@unpad.ac.id (Y.H.); 5 Department of Pharmaceutics, Faculty of Pharmacy, Minia University, Minia 61519, Egypt; ahmed.mohamed1@minia.edu.eg; 6 School of Pharmaceutical Sciences, Universiti Sains Malaysia, Gelugor 11800, Penang, Malaysia; amirahmg@usm.my; 7 Physiology Division, Department of Anatomy, Physiology and Biology Cell, Faculty of Medicine, Universitas Padjadjaran, Sumedang 45363, Indonesia; ronny@unpad.ac.id; 8 Departement of Physics, Faculty of Mathematics and Natural Sciences, Universitas Padjadjaran, Sumedang 45363, Indonesia

**Keywords:** alpha mangostin, nanoparticles, DMBA, in vivo, breast cancer

## Abstract

α-mangostin (*Amg*), a compound isolated from the mangosteen rind (*Garcinia mangostana,* L.), has demonstrated promising anticancer activity. However, its low solubility and selectivity against cancer cells limit its efficacy. To address this issue, researchers have developed chitosan/alginate polymeric nanoparticles (NANO-AMCAL) to enhance the effectiveness of *Amg*. In vitro studies have demonstrated that NANO-AMCAL is highly active against breast cancer cells. Therefore, an in vivo study was conducted to evaluate the efficacy of NANO-AMCAL in treating breast cancer in Wistar rats (*Rattus norvegicus*) and determine the effective dose. The rats were divided into seven treatment groups, including positive control, negative control, pure *Amg*, and NANO-AMCAL 5 mg, 10 mg, and 20 mg. The rats were injected subcutaneously with a carcinogenic agent, 7,12-dimethylbenz(a)anthracene (DMBA) and were evaluated for weight and tumor volume every three days during treatment. Surgery was performed on day 14, and histopathological studies were carried out on breast and lung cancer tissues. The results showed that NANO-AMCAL significantly enhanced the anticancer activity of *Amg* in treating breast cancer in Wistar rats. NANO-AMCAL containing 0.33 mg of *Amg* had a healing effect three times better than 20 mg pure *Amg* and was comparable to tamoxifen. The effective dose of NANO-AMCAL for anti-breast cancer treatment in Wistar rats was found to be 20 mg, which exhibited a good healing response, and the tumor volume continued to decrease up to 17.43% on the 14th day. Furthermore, histopathological tests showed tissue repair and no metastases. These findings suggest that NANO-AMCAL may be a promising therapeutic option for breast cancer treatment.

## 1. Introduction

Cancer remains a major health problem in the world, with breast cancer being the most prevalent type among women [1]. As a metastatic cancer, it can spread to other organs, such as the brain, liver, bones, and lungs, leading to a higher mortality rate [2]. Malignant tumors can invade the surrounding tissues and spread to other parts of the body [3]. Currently, the treatment options for breast cancer include chemotherapy, surgery, radiotherapy, and hormonal therapy [4]. The selection of treatment depends on the stage, level of cancer cells, and type [5]. However, some of these therapies can have severe side effects, including damage to healthy cells, which can be life-threatening [6,7]. Therefore, alternative treatment options that are safer and more effective are urgently needed [8,9].

Mangosteen (*Garcinia mangostana*. L.) is known to contain α-mangostin (*Amg*) [10], which has been found to possess anticancer activity [11]. However, the poor solubility of *Amg* and its low selectivity to cancer cells limit its potential as an effective cancer treatment [12,13,14]. To overcome these challenges, previous studies have focused on developing a nanoparticle-based formulation of *Amg*. Chitosan alginate polymeric nanoparticles (NANO-AMCAL) have been formulated and tested for their efficacy in vitro [15]. The combination of chitosan and alginate has shown great promise to improve drug delivery in cancer therapy [16]. Chitosan acts as a dissolving agent and drug stabilizer and enables controlled drug release [17]. It also provides a multifunctional platform for targeting and stimulus-responsive release and can be used in image-guided medicine [17,18]. On the other hand, alginate offers advantages such as biocompatibility, controlled drug release, targeting abilities, enhanced cellular uptake, and reduced systemic toxicity [19,20]. Combining chitosan and alginate may create a synergistic effect, resulting in more efficient and targeted delivery of drugs, including potential anticancer agents, like *Amg* [21].

In the pharmaceutical field, two primary types of nanoparticles commonly described are nanocrystals and nanocarriers [22]. Nanocrystals are drug particles that have been reduced in size to the nanometer range, while nanocarriers are specific carrier systems that are used to encapsulate drugs. Reduction in the particle size of a drug can improve its solubility and increase the overall surface area, which could enhance the efficacy and bioavailability of drugs [23,24]. Nanoparticles also offer several other advantages, including targeted drug delivery, reduced toxicity, and improved stability [25].

In this study, the in vivo effectiveness of NANO-AMCAL as an anti-breast cancer agent in DMBA-induced Wistar rats (*Rattus norvegicus*) was evaluated, and its optimal dosage was determined. This evaluation involves administering different dosages of NANO-AMCAL to the rats and monitoring their weight and tumor volume during treatment. The results of this treatment assist in determining the optimal NANO-AMCAL dosage required for the effective treatment of breast cancer and assessing its safety for future human use.

## 2. Materials and Methods

### 2.1. Materials

#### 2.1.1. Chemicals

α-mangostin 98% (*Amg)* (Biopurify Chengdu^®^_,_ China), Chitosan pharmaceutical grade (300 kDa), and alginate was purchased from Interlab, Ltd. (Jakarta, Indonesia).

Dimethylbenz[a]anthracene (DMBA), tamoxifen, and corn oil were purchased from Sigma Aldrich (St. Louis, MO, USA); ethanol, tween 80, 0.9% sodium chloride, tripolyphosphate (TPP), rat food, atrigel, ether, NaCMC, and formalin were purchased from (Merck, Boston, USA); and liquid nitrogen was purchased from (Bratachem^®^, Indonesia). 

#### 2.1.2. Cells

Cell line MCF7 was obtained from the Laboratory of Cell Culture Central Laboratory (**ATCC** HTB-22), Universitas Padjadjaran. MCF7 cells were prepared in an RPMI medium 24 h before the experiment in a 24-well plate. 

### 2.2. Animals and Foods

Female Wistar rats (*Rattus norvegicus*) with body weights of more than 200 g and ages of more than 10 weeks were obtained from the Bandung Institute of Technology, Indonesia. The animals were acclimatized for one week after procurement and fed using standard rodent pellet feed and water ad libitum during the experimental period [23,26].

### 2.3. Preparation of NANO-AMCAL DDS 

The NANO-AMCAL formula has been registered as a patent at the Indonesian Patent Office No. P00202304573 [27]. NANO-AMCAL was formulated using the ionic gelation method. The method has been briefly described in the literature [28,29]. Table 1 shows the formula of NANO-AMCAL.

**Figure 1:**
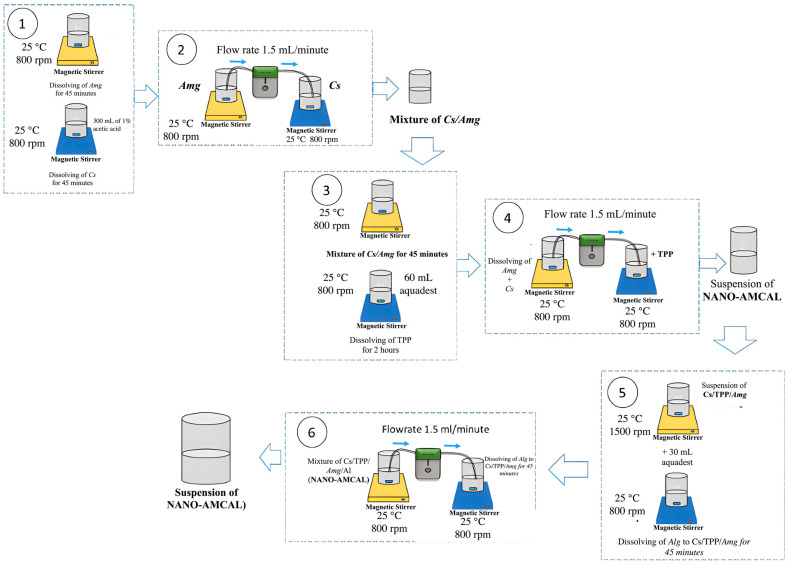
Preparation of NANO-AMCAL.

**Figure 2:**
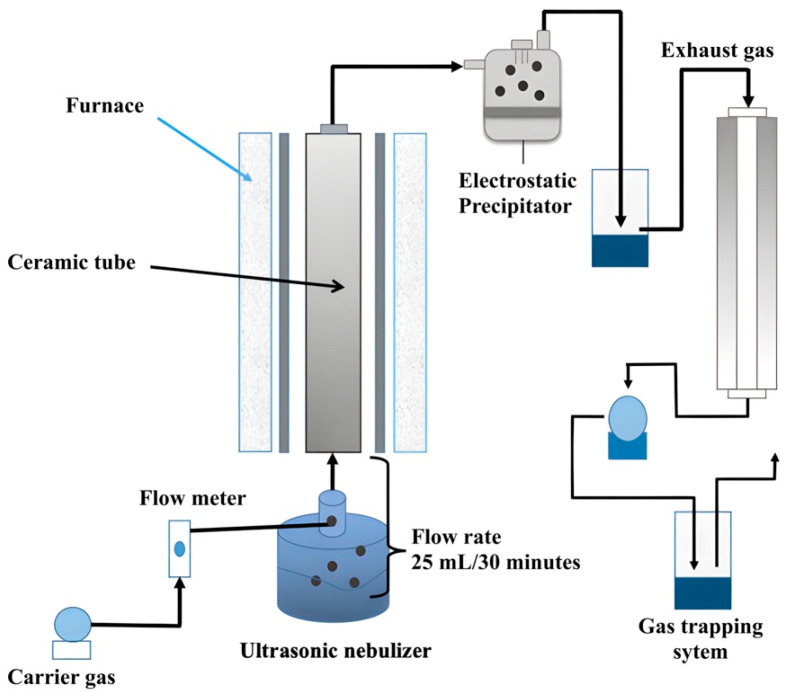
Ultrasonic-assisted spray dryer.

Figure 1 depicts the process of preparing *Amg* drug delivery system (DDS) using an ultrasonic-assisted spray drying method (Figure 2). The process begins by dissolving chitosan (Cs) powder (0.3 g) in 300 mL of 1% acetic acid using a magnetic stirrer for two hours at room temperature (25 °C) and a stirring speed of 800 rpm. Separately, a solution of *Amg* (0.03 g) was prepared by dissolving in 30 mL of 96% ethanol at room temperature (25 °C) with a stirring speed of 1500 rpm (Step #1). The *Amg* solution was then added dropwise to the chitosan solution using a micropump with a flow rate of 1.5 mL/min while continuously stirring for two hours (Step #2). Then, a TPP solution was prepared by dissolving 0.06 g TPP in 60 mL of distilled water at room temperature and adding distilled water until the total volume reached 300 mL (Step #3). Finally, the Cs/*Amg* solution was transferred dropwise into the TPP solution prepared in Step #3, producing a suspension of Cs/TPP/*Amg* nanoparticles (Step #4). 

The addition of alginate in Step #5 allowed the creation of NANO-AMCAL. Alginate solution was prepared by dissolving 0.03 g of alginate in 30 mL of distilled water at varying concentrations of 10, 25, and 50 wt.% of chitosan to investigate the effect of alginate concentration on the final product. The *CS/AMG* nanoparticles obtained from Step #4 were transferred dropwise into the alginate solutions to produce NANO-AMCAL (Step #6).

To obtain a dry powder of NANO-AMCAL, the suspension was subjected to spray-drying using an ultrasonic-assisted spray drying system, as shown in Figure 2. In this technique, the suspension of NANO-AMCAL was atomized by an ultrasonic wave to form droplets of NANO-AMCAL that passed through the ceramic tubular inside the furnace with the aid of a continuous flow of carrier gas (O_2_) in the chamber. The water molecules that are present in the droplets evaporate during their residence time in the furnace, leading to the production of dried particles that are collected at the end of the process. This technique has been previously reported to be efficient in producing nanoparticles with high drug-loading capacity and controlled release properties [30,31]. 

### 2.4. Physicochemical Characterization of NANO-AMCAL 

The nanoparticles of NANO-AMCAL were characterized in terms of particle size, size distribution, and surface charge using a particle size and zeta potential analyzer (SZ 100 Horiba). The functional groups and intermolecular interactions present in the nanoparticles were analyzed using FTIR spectroscopy (Thermo Nicolet iS5). The morphology, particle size, and shape were observed using both a scanning electron microscope, SEM (Hitachi SEM SU3500), and a transmission electron microscope, TEM (HR TEM H9500). Additionally, the crystallinity was determined using X-ray diffraction (XRD) (Bruker D8 Advance). 

### 2.5. Evaluation

#### 2.5.1. Entrapment Efficiency (%)

To measure the entrapment efficiency, the amount of untrapped *Amg* was indirectly measured. The Cs/*Amg*/Alg nanoparticle suspension was subjected to centrifugation at 3000 rpm for 10 min, following which the supernatant was extracted and separated from the pellet. The total amount of *Amg* was calculated by multiplying the initial concentration of *Amg* used in the preparation of nanoparticles by the volume of the nanoparticle suspension, and the amount of untrapped *Amg* was determined by subtracting the amount of *Amg* present in the pellet from the total amount of *Amg* used in the preparation of the nanoparticles, using UV/Vis spectroscopy (Analytic Jena Specord 200) at the maximum absorption wavelength of 245 nm. The entrapment efficiency was calculated using Formula (1):

(1) $$Entrapment\;efficiency\;\left(\%\right)=[1-\frac{Amount\;of\;untrapped\;\alpha -mangostin\;}{Total\;amount\;of\;\alpha -mangostin}\times 100]$$

#### 2.5.2. Solubility Test

A saturation solubility test was conducted to evaluate the solubility of pure drugs, Cs/TPP/*Amg*, and NANO-AMKAL in distilled water. In this experiment, 15 mg of the tested substance was introduced in 5 mL of distilled water (pH of 7.4), and the mixture was constantly stirred for 24 h to ensure complete dissolution. The resulting solution was then filtered and diluted in a 1:3 ratio, and then the concentration of *Amg* using a UV/Vis spectrophotometer at a wavelength of 245 nm was analyzed [32]. 

#### 2.5.3. In Vitro Release Profile

The in vitro release study of *Amg* from NANO-AMCAL was conducted under three different pH conditions, pH 1.2, pH 5.0, and pH 7.4 to simulate the varying pH levels in the gastrointestinal tract. At each time interval (5, 10, 15, 30, 45, 60, and 120 min), 5 mL of buffer was collected, and an equal volume of fresh buffer was replenished immediately. The concentration of Amg in the buffer solution was analyzed at 245 nm using a UV-Vis spectrophotometer to calculate the cumulative percentage of substance release from the NANO-AMCAL (n > 3) [32]. Nanoparticles can enhance α-mangostin delivery, such as via pH-responsive behavior [33]. (1) Protonation of the cationic polymer and chitosan will allow the transformation of NANO-AMCAL in different pH environments. (2) The encapsulation of α-mangostin in NANO-AMCAL nanoparticles will protect *Amg* from degradation, improve its stability, and prevent its premature release. (3) The pH-responsive behavior of NANO-AMCAL allows the controlled and targeted release of *Amg*. In an acidic environment, such as the tumor microenvironment, the protonation of chitosan will cause swelling and disintegration of the nanoparticles, facilitating the sustained and controlled release of *Amg*. (3) The small size and positive surface charge of NANO-AMCAL can facilitate cellular uptake. Positively charged nanoparticles can interact with negatively charged cell membranes, promoting their internalization via endocytosis. (4) NANO-AMCAL provides a protective barrier around *Amg*, protecting it from enzymatic degradation and metabolic processes. This protection helps maintain the stability and bioactivity of *Amg* during its delivery to the targetted site [17].

#### 2.5.4. Cytotoxicity Assay (MTT Assay)

The MTT assay was performed to assess the cytotoxicity of the nanoparticles on MCF7 cells. Briefly, MCF7 cells were seeded at a density of 1 × 10^4^ cells/mL in a 96-well plate and incubated overnight. Subsequently, the cells were treated with different concentrations of NANO-AMCAL (0, 3.125, 6.25, 12.5, 25, 50 μg/mL) in DMEM medium containing 1% FBS and incubated for 24 h at 37 °C in a CO_2_ incubator under dark conditions. After the incubation, 10 μL of an MTT solution (5 mg/mL) was added to each well, and the cells were further incubated for 4 h at 37 °C. Next, the media were removed, and 100 μL of SDS was added and agitated to dissolve the purple formazan crystals. The absorbance of each well was measured at 570 nm, with 630 nm as the reference wavelength. The percentage of cell viability was calculated by comparing the absorbance of the treated cells to the control cells [32,33].

### 2.6. Preparation of Test Animals

The preparation of test animals for experimentation involves several stages [16].

First, healthy female Wistar strain rats (*Rattus norvegicus*) with body weights over 200 g and ages over 10 weeks were selected. The rats were then grouped into seven groups, with each group consisting of four rats. The grouping of the test animals is presented in Table 2.

The animals were allowed to acclimatize to the laboratory conditions for one week prior to the commencement of the experiment. The laboratory environment was maintained at a constant temperature of 22 ± 3 °C and a humidity of 50 ± 10%, with 12:12 h of light and dark cycles. Throughout the study period, tumor volume was measured every three days for 21 days following tumor induction. The measurement was carried out using a caliper to determine the diameter of the largest and smallest tumors [17].

### 2.7. DMBA Induction

For the DMBA induction, 25 mg of DMBA was diluted with 0.5 mL of corn oil and sodium chloride (0.9% NaCl) using a sterile syringe for single-dose injection. The injections were administered subcutaneously into the breasts of mice [15]. The mice were then monitored every three days for the growth of breast tumors, as well as changes in body weight and tumor volume until day 21, by which time the tumors should reach a size of approximately 200–400 mm^3^ [34,35,36].

Tumor volume is calculated using Formula (2) [37]:

Tumor volume = 0.5 (l × w) (2)

Once the tumor reached the target size, the rats were divided into 7 treatment groups, as specified in Table 2. This is in accordance with the Frederer formula:

Number of test animals = (n − 1) (t − 1) ≥ 15 (3)

* n = the sample size for each intervention and t = the number of interventions [38].

Each group had 4 repetitions. During the treatment duration, weight and tumor size were measured every 3 days until day 14, after which the animals were sampled for further tests.

### 2.8. Histopathology

The test animals were euthanized on day 14, and their breast and lung tissue were extracted and fixed in 10% neutral buffered formaldehyde for 24 h. The fixed tissues were dehydrated using an ascending series of alcohol and stored in a 1:1 alcohol–water mixture before being incubated in laboratory-grade benzene for 1 h. The tissue pieces were then embedded in paraffin wax, cut into 5 mm blocks, mounted on glass slides, and stained with hematoxylin and eosin (H&E). The slides were then viewed under a light microscope for further analysis [23,38,39].

### 2.9. Statistical Analysis

Statistical analysis was performed on the tumor volume measurements and rat body weight data. The Shapiro–Wilk test was used to assess whether the data were normally distributed. If the data exhibited a normal distribution, analysis of variance (ANOVA) was performed using the SPSS application.

## 3. Results and Discussion

### 3.1. Characteristics of NANO-AMCAL Suspension 

**Figure 3:**
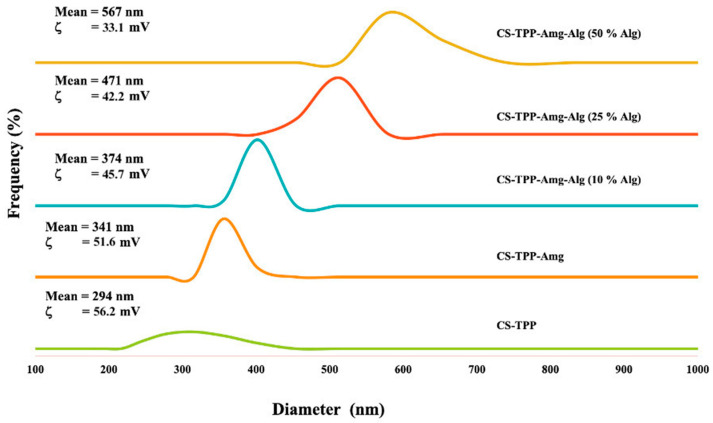
Zeta potential of Cs/TPP/*Amg*/Alg nanoparticles.

The findings regarding the particle size and zeta potential of the nanoparticles are presented in Figure 3. It was observed that in general, an increase in the concentration of alginate resulted in an increase in nanoparticle size, whereas the surface charge showed an inverse trend. Previous studies have reported that the use of alginate in nanoparticle coatings can lead to an increase in particle size, and the degree of increment is dependent on the concentration of alginate used in the formulation [40]. For drug delivery applications, nanoparticles, which are known as submicroscopic colloidal drug carrier systems, will have sizes of between 10 and 400 nm. This size range is desirable for drug carriers because they have the advantage of being able to carry large amounts of drugs [41,42]. 

The size of NANO-AMCAL is affected not only by the concentration of alginate but also the viscosity of the alginate solution [40]. The viscosity of the solution can influence the diffusion of droplets, which can impact the final size of the nanoparticles. The increase in particle size is mainly due to the presence of manuronate and guluronate [43], which are the two primary monomers that make up alginate. These monomers form a polymer chain that contributes to the thickness of the alginate coating around the nanoparticles. Additionally, the surface charge of the particles decreases with increasing alginate concentration due to the anionic nature of alginate. Chitosan, on the other hand, is a polymer with a highly positive surface charge; thus, the presence of alginate coating neutralizes the positive surface charge of the nanoparticles, which ultimately reduces the overall values [44,45].

### 3.2. Characterization of NANO-AMCAL 

#### 3.2.1. Fourier-Transform Infrared (FTIR)

**Figure 4:**
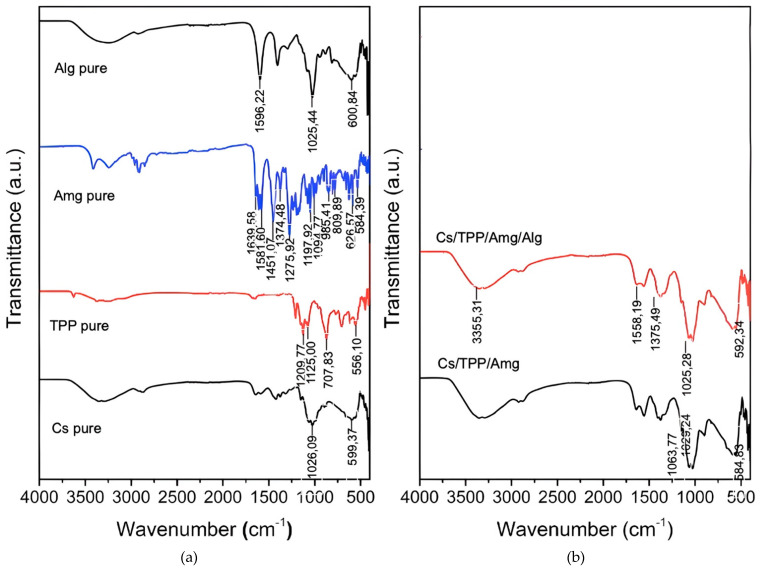
The FTIR spectra of (**a**) raw materials in comparison to (**b**) the NANO-AMCAL, Cs/TPP/Amg of nanoparticles.

Figure 4a shows the FTIR spectrum of the raw materials and Figure 4b shows the FTIR spectrum of the two nanoparticles: NANO-AMCAL and Cs/TPP/Amg. Both nanoparticles exhibited C-O stretching at 1029.24 cm^−1^, C-H stretching at 2948.21 cm^−1^, and O-H/N-H stretching at 3358 cm^−1^. Chitosan and alginate polymers have spectral similarities in the functional analysis of chitosan and alginate because chitosan and alginate have similar functional groups due to the existence of –NH2 and –OH in both polymers. The functional group analysis showed the interaction of polyelectrolytes between the -COO^−^ group of alginate and the -NH2 group of chitosan, which is characterized by the appearance of vibrations in the wavenumber 1400 cm^−1^ [46]. The peak intensity of Cs/TPP/Amg nanoparticles at 3348 cm^−1^ changes with the addition of alginate polymers [47], 3358 cm^−1^ (alginates 10%), 3355 cm^−1^ (alginates 25%), and 3284 cm^−1^ (alginates 50%), in addition to the presence of additional peaks between 1375 cm^−1^ and 1558 cm^−1^ at all alginate concentrations used. These changes in spectrum pattern also confirm the presence of alginate coating on the surface of Cs/TPP/Amg nanoparticles. 

#### 3.2.2. Crystalinity 

**Figure 5:**
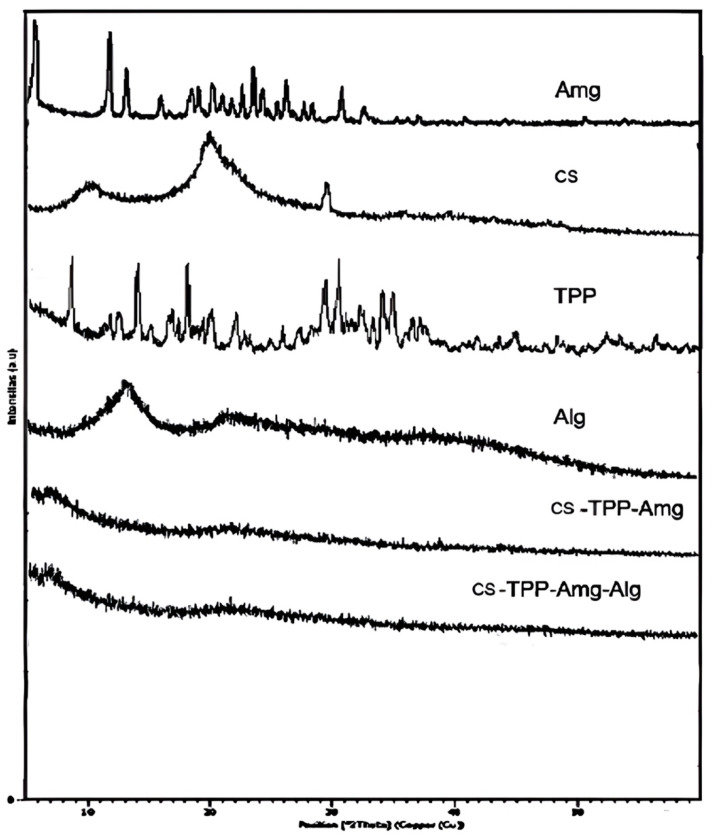
The XRD pattern of NANO-AMCAL in comparison to the raw materials.

Figure 5 presents the diffractograms of NANO-AMCAL and the raw materials used. XRD measurements were conducted at an angle of 5–60 θ, and the results showed that both *Amg* and TPP are crystalline materials. The highest peak of *Amg* was observed at 7°–32°, while TPP was found at 30.4376°. Chitosan exhibited three peaks at 10.4550°, 19.7187°, and 29.3342°, indicating its semi-crystalline nature, while alginate had a peak at 13.3798° [48]. The formation of nanoparticles altered the semi-crystalline nature of the biopolymers to amorphous nanoparticles, as evidenced by the XRD spectrum of the Cs/Amg and NANO-AMCAL. This is in agreement with the finding by Jampafuang et al., who described that the crystalline structure of chitosan is fully destroyed following crosslinking with TPP, producing amorphous nanoparticles [49]. 

#### 3.2.3. Morphology 

**Figure 6:**
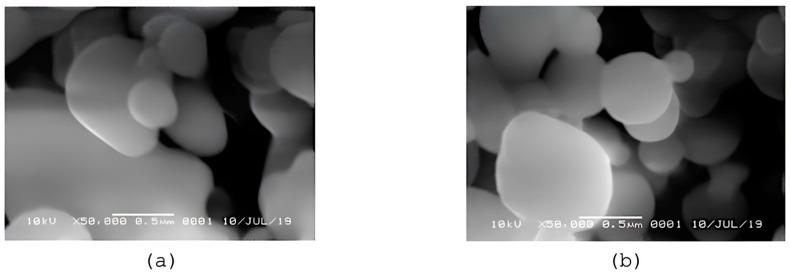
The SEM images with 50,000 times magnification on nanoparticles: (**a**) CS-TPP-Amg, (**b**) NANO-AMCAL.

**Figure 7:**
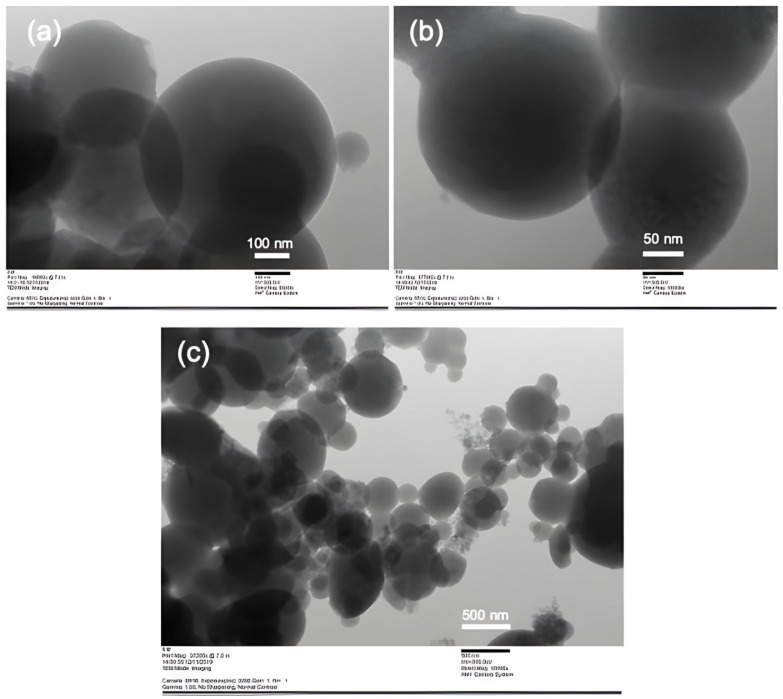
TEM images of NANO-AMCAL at different magnifications. (**a**) Particle sizes of 100 nm, (**b**) Particle sizes of 50 nm, (**c**) Particle sizes of 500 nm.

Figure 6 displays the morphology of Cs/TPP/Amg and NANO-AMCAL obtained from SEM analysis. In general, the nanoparticles have a round shape, but the shape becomes more irregular with increasing alginate coating concentration. Figure 7 shows the TEM images of the nanoparticles with different magnifications, which appear as spherical vesicles with fewer dense regions surrounding them, indicating the presence of a coating layer around the chitosan nanoparticles.

**Figure 8:**
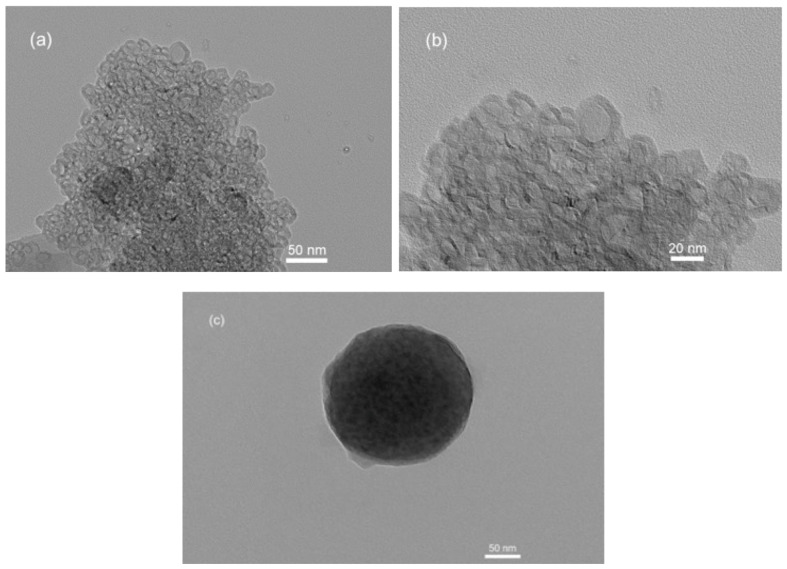
TEM images of NANO-AMCAL with alginate 10% under different magnifications. (**a**) Aggregated particle sizes of 5 nm–50 nm, (**b**) Particle sizes of 20 nm, (**c**) Particle sizes of 50 nm.

Additionally, Figure 8 shows nanoparticles without alginate polymer with particle sizes of 5 nm and 50 nm, and the perfectly round particle can be observed in the picture. The TEM images in Figure 8 also display the Cs/TPP/Amg nanoparticles with particle sizes of 50 nm and 20 nm, respectively. It is apparent that when the particles are coated with alginate polymers, previously aggregated particles become separated. Moreover, there are noticeable differences in color between the inside and the outside of the particles, where the inner part appears crystal clear, while the outer part is black.

Therefore, these TEM results provide further evidence of the nature of water-soluble alginate polymers, which enhance the solubility of *Amg* compounds.

### 3.3. In Vitro Evaluation of NANO-AMCAL

#### 3.3.1. Results of Entrapment Efficiency

The entrapment efficiencies (%) and loading capacity (%) of *Amg* in the nanoparticles are presented in Table 3. It can be observed that the entrapment efficiency decreases with the increasing concentration of alginate used in the process.

#### 3.3.2. Results of In Vitro Release Profile

**Figure 9:**
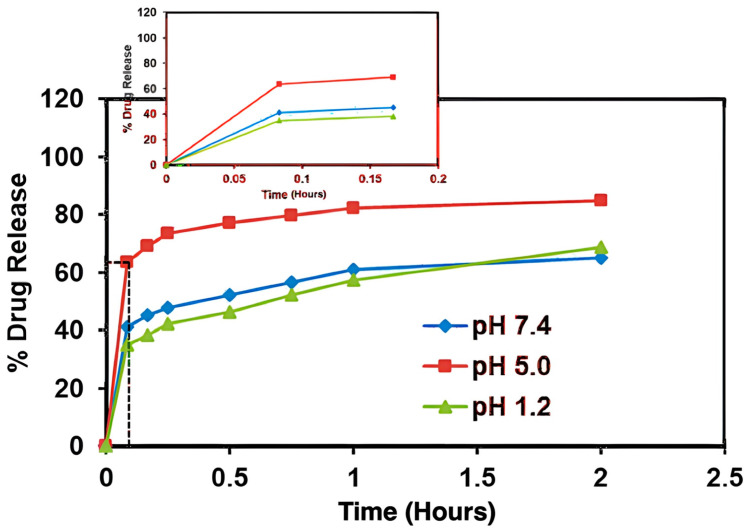
Drug release performances of NANO-AMCAL 10 wt.% at various pH conditions.

The release profile of *Amg* from the NANO-AMCAL is found to be pH-dependent (Figure 9). It is released much faster at pH 5.0 compared to pH 1.2 and 7.4. The alginate in the nanoparticle coating is capable of withstanding the acidic condition of pH 1.2 due to the presence of glucuronate content that forms a strong gel that retards the release of the encapsulated content [34]. 

At pH 5.0, the chitosan network expands more, leading to a faster drug release than that observed at other pH values [50,51]. Based on this finding, it is evidenced that these nanoparticles could be tailor-made for specific functions. A fast-release nanoparticle could be developed for drugs intended to be released in the duodenum (pH 5–6) following oral delivery, while a slower release may be possible if the loaded drug is meant to be delivered into the bloodstream. Moreover, a targeted release could be achieved in the cancer environment, which is known to be more acidic than the normal cellular environment [33]. 

#### 3.3.3. Results of Solubility

**Figure 10:**
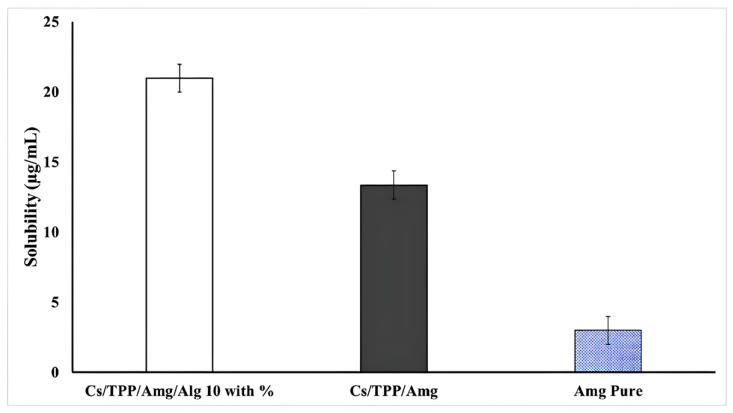
The solubility of NANO-AMCAL 10 wt.% in comparison with Cs/TPP/Amg (without alginate) and pure Amg.

In this study, a solubility test was conducted for 24 h using water as a solvent to assess the ability of nanoparticles to increase the solubility of *Amg*. As shown in Figure 10, a significant difference was observed in pure *Amg*, Cs/TPP/*Amg,* and NANO-AMCAL. 

Despite containing several -OH groups, *Amg* is known to have low aqueous solubility due to its high carbon content, which renders the molecules more hydrophobic and less polar as the number of carbon atoms increases. The formulation of *Amg* into alginate-coated chitosan nanoparticles has significantly increased its water solubility. Although chitosan is not soluble in aqueous media at neutral pH, it can dissolve in a slightly acidic environment due to the presence of amine groups. However, the increase in solubility is likely due to the outer coating of alginate, which dissolves easily in water.

#### 3.3.4. Cytotoxicity Assay

*Amg* has been reported to exhibit potent anticancer activity against breast cancer [52]. It stimulates a stable protective effect against TPA-mediated metastasis. The antimetastatic effect of *Amg* on TPA-induced MCF7 cells is due to the inactivation by 1/2 extracellular signals (ERK1/2) and the reduction in AP-1 and NF-Kb DNA binding activity, which leads to a reduction in the expression of MMP-2 and MMP-9 [53]. It is noteworthy that in this study, the effectiveness of *Amg* was found to be higher in the form of nanoparticles, and the presence of alginate coating increases the effectiveness compared to uncoated chitosan nanoparticles.

**Figure 11:**
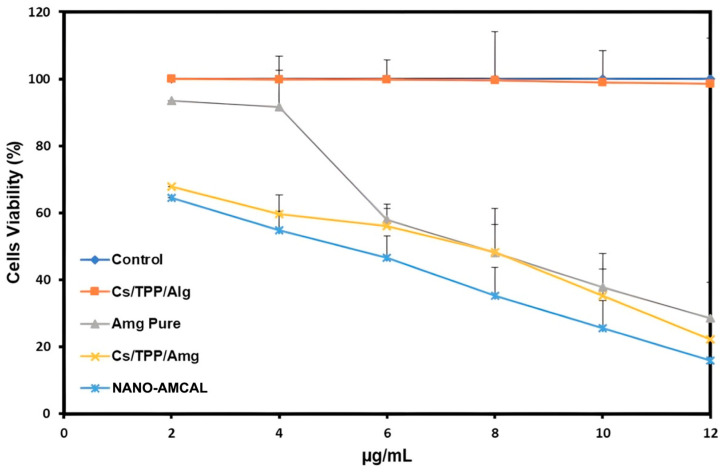
Cell viability of MCF7 after treatment with NANO-AMCAL 10%.

Figure 11 illustrates the cell viability following treatment with both *Amg* (pure) and the 10% NANO-AMCAL. The use of 10% Alg concentration was selected, as it exhibited the best overall characteristics in terms of particle size and entrapment efficiency. The IC_50_ values are presented in Table 4. Notably, increased cytotoxicity was observed following the formulation of *Amg* in the form of NANO-AMCAL, which may be attributed to the enhanced cellular uptake of the *Amg*-loaded chitosan nanoparticles. Chitosan nanoparticles are positively charged, which leads to their electrostatic attraction to the negatively charged cell surface, thus facilitating higher cellular uptake. Once the *Amg* successfully enters the cells, it can disrupt the cellular components by damaging the DNA, resulting in DNA fragmentation and, ultimately, cell death [54].

A further decrease in IC_50_ value was observed following the treatment with NANO-AMCAL as shown in Table 4. The application of alginate for the encapsulation of anticancer drugs not only can lead to controlled and sustained drug release but also highly improve the effectiveness of anticancer drugs against cancers by causing damage to the cells by reducing the intracellular concentration of copper ions. These copper ions are crucial in the defense and proliferation of cancer cells, and their removal can affect important cellular processes, such as respiration and maturation. In addition, the presence of alginate can improve the stability of nanosystems in the acidic environments of biological fluids [55]. 

### 3.4. In Vivo Evaluation

#### 3.4.1. DMBA Induction

**Figure 12:**
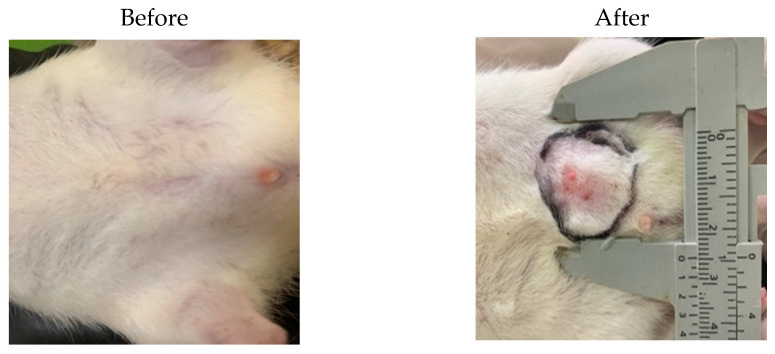
Results of DMBA Induction.

Chemical induction using dimethylbenz[a]anthracene (DMBA) was performed to induce cancer in experimental animals. The mechanism of DMBA as a breast cancer induction agent is through rapid interaction with proliferating cells in the terminal end bud to form DNA adducts, which play a crucial role in transforming normal end bud cells into cancer [24]. The initial step involved selecting female white Wistar rats with a body weight of 200 g and a maximum age of 6 months. Afterward, the animals underwent a quarantine or environmental adjustment for 7 days to ensure a stable and controlled environment for the experiment. The rats were divided into seven groups: normal (without DMBA induction), control, tamoxifen, *Amg* Pure (P.Amg), NANO-AMCAL 5 mg, NANO-AMCAL 10 mg, and NANO-AMCAL 20 mg, where each group consisted of four rats. Afterwars, induction was carried out in six groups, except the normal group. A single-dose injection solution of 7,12-dimethylbenz[a]anthracene (DMBA) dissolved in 0.5 mL corn oil and 0.5 mL sodium chloride 0.9% was prepared and homogenized using a vortex homogenizer. The rats were then subcutaneously injected with DMBA into the breast tissue, and an initial evaluation was performed by weighing their body weight and subsequent body weight every 3 days as shown in Figure 12. Once the tumor was formed in all rats, changes in tumor volume were measured in the first week along with body weight measurments every 3 days for 21 days. A morphological assessment was used to confirm that the lump was cancerous tissues (certificial tumor). Excessive cell development causes the formation of a cancerous lump that forms solid aggregates. If left untreated, the lump will continue to grow in size until it finally breaks [25]. In the first week, a tumor formed with an average volume of 78 mm^3^. 

#### 3.4.2. NANO-AMCAL In Vivo Testing (NPαM)

On Day 21 after induction, five groups were orally administered with the respective treatment: the positive control group using tamoxifen and the P. *Amg* group using NANO-AMCAL 5 mg, NANO-AMCAL 10 mg, and NANO-AMCAL 20 mg. The treatment doses administered to the test animals are summarized in Table 5.

**Figure 13:**
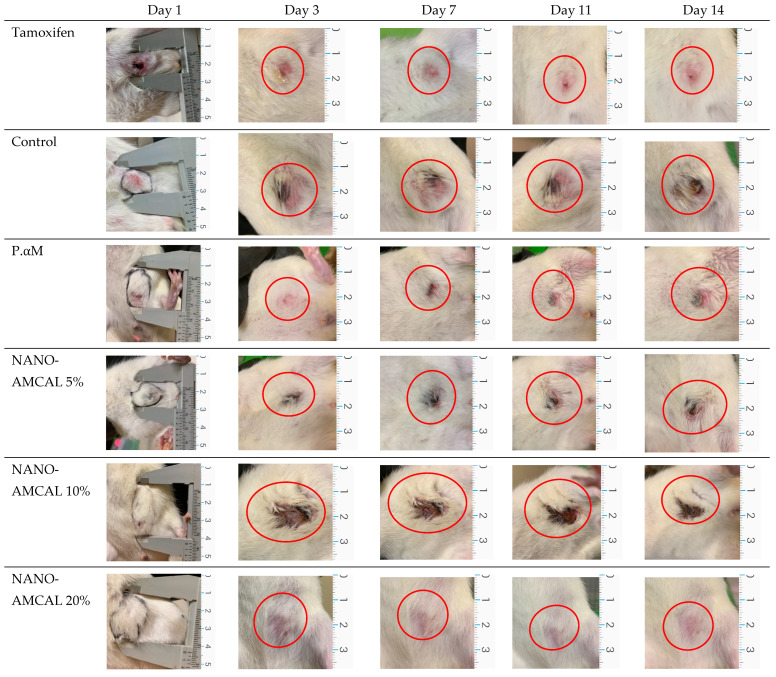
Results of the therapeutic treatment of NANO-AMCAL. The red circle was shown the tumor condition after treatments.

To assess the effectiveness of the treatments, a control group was included, which was induced with DMBA but did not receive the test preparation. This control group served as a reference to compare the tumor conditions with and without the treatment. Body weight and tumor volume were measured for all groups on days 0, 3, 7, 11, and 14. The results of the therapeutic treatments are presented in Figure 13.

Evaluation results include:

1. Observation Results of Rat Body Weight

**Figure 14:**
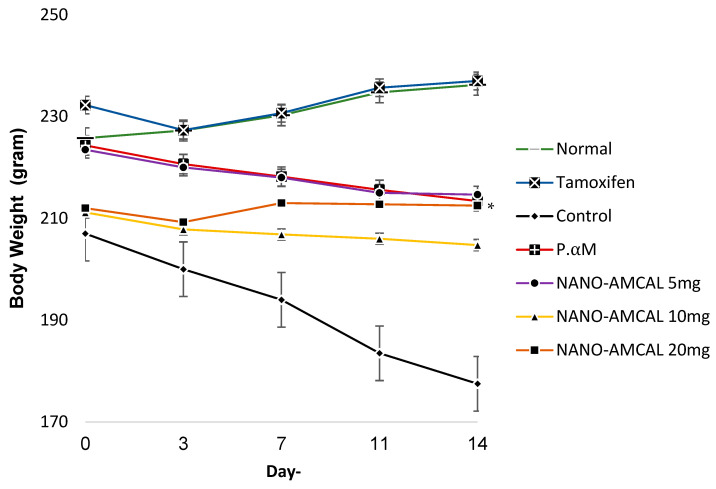
Changes in body weight of the test animals (n = 4), * *p* < 0.05 for NANO-AMCAL preparations.

Body weight measurements were recorded on days 0, 3, 7, 11, and 14 to monitor the effects of the treatment on the test animals. Figure 14 showed that the control group exhibited a significant and rapid weight loss compared to the groups receiving test preparations from day 0 to day 14. Notably, the P.αM group exhibited better control of body weight than the control group. Moreover, the nanoparticle groups displayed superior efficacy in regulating the body weight of the western rats, with the NANO-AMCAL 20 mg demonstrating no significant difference (*p* < 0.05) compared to the tamoxifen group.

The ANOVA test using Duncan’s post hoc analysis revealed that the NANO-AMCAL 20 mg group was the most effective dose in controlling body weight, followed by the 10 mg and 5 mg doses. Additionally, NANO-AMCAL can downregulate Bcl-2 expression, with higher doses resulting in more substantial reductions in expression, as indicated by a more stable body weight graph.

2. Observation Result of Tumor Volume

**Figure 15:**
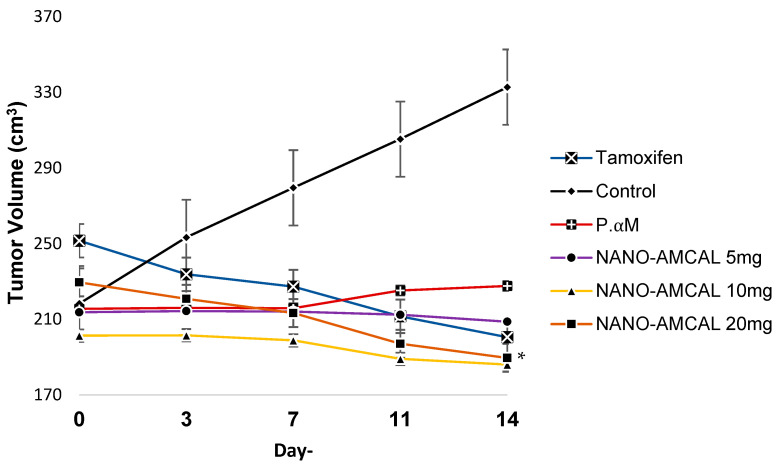
Growth of tumor volume in the test animals (n = 4), * *p* < 0.05 for NANO-AMCAL preparations.

Figure 15 shows the results of the tumor volume measurement showed that in the negative control group, tumor volume continued to increase significantly from day 1 to day 14. However, in the pure *Amg* group, the tumor volume tended to be stable in the first week and experienced a slight increase in the second week. The NANO-AMCAL test preparation at a dose of 20 mg exhibited a decrease in tumor volume that was not significant compared to the tamoxifen group (*p* > 0.05), indicating no significant difference between the two groups. The graph of the tumor volume growth of the test animals is presented in Figure 15.

3. Results of the Tumor Histology Analysis

**Figure 16:**
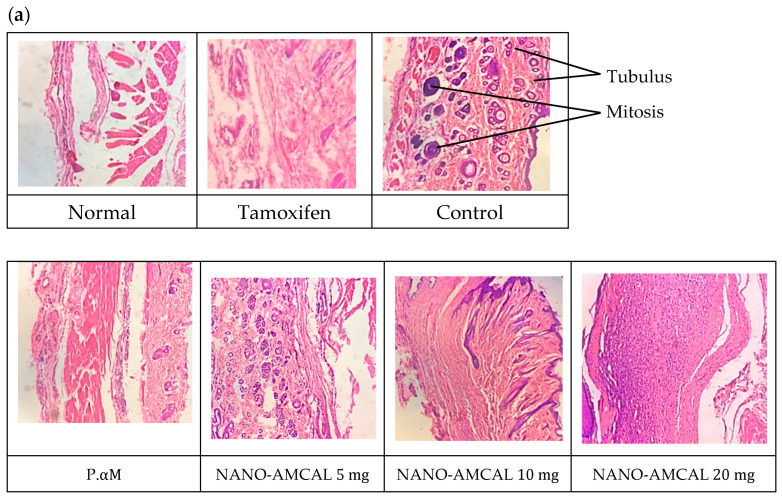

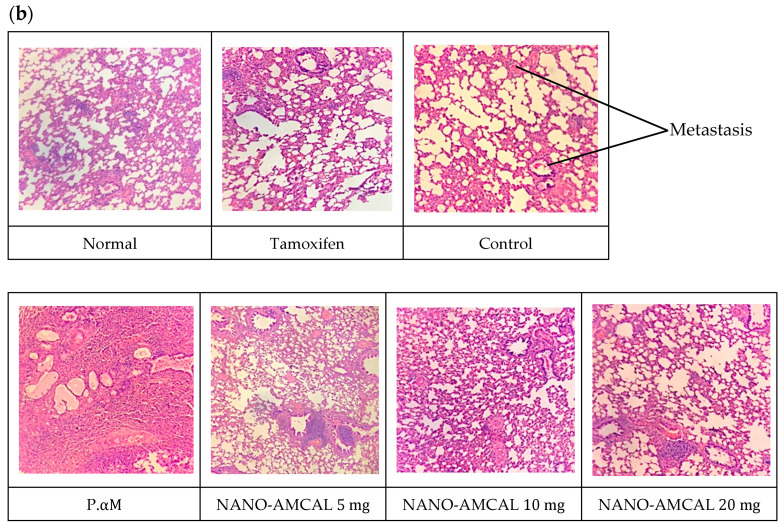
(**a**) Histology of the tumor; (**b**) histological appearance of the lung (All images are magnified up to 40×).

**Figure 17:**
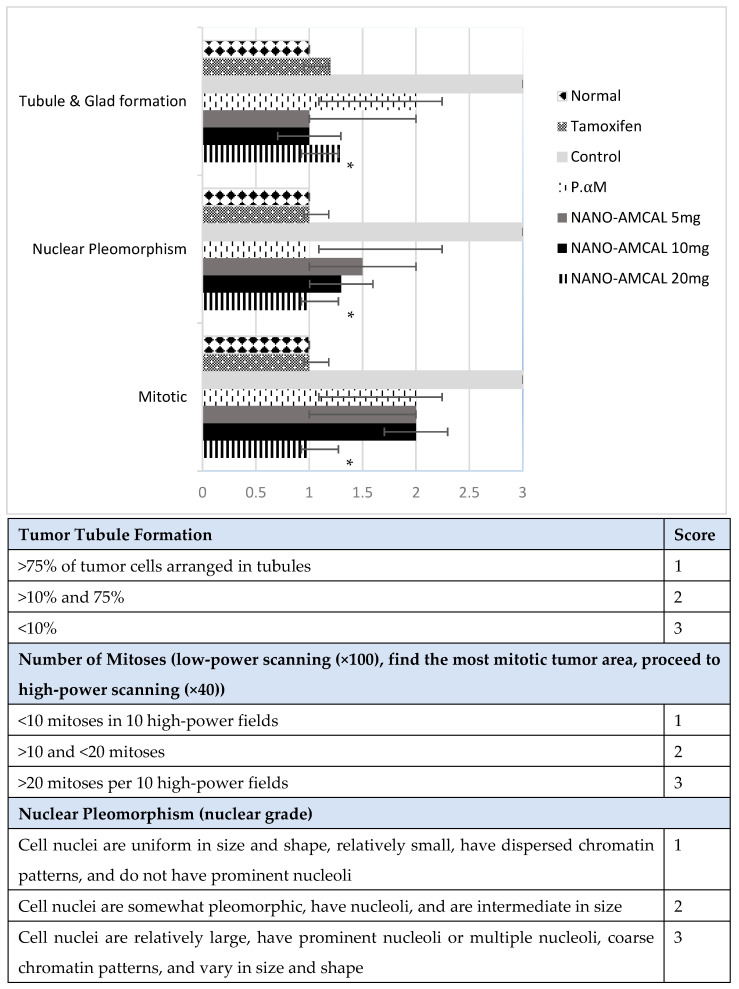
Histopathological grading results (n = 4); * *p* < 0.05 for NANO-AMCAL preparations.

After day 14 of therapy, the rats were euthanized under inhalation anesthesia using ether, and the breast tumor and lung tissues were collected for further analysis. To preserve the cellular morphology and chemical integrity of the tissues, they were fixed in 10% neutral buffered formalin. This process helps to maintain the tissue structure and prevent damage from decay and tissue migration. After fixation, the tissues were subjected to H&E staining and examined microscopically to observe any changes in cellular morphology and tissue structure. Figure 16 represents the results obtained from the H&E staining of the tissue samples, and Figure 17 describes the histopathological grading of breast tumor tissue.

In a study conducted by Ting Yu (2019), *Amg* was administered in the form of a polymeric nanoparticle called PECE at a dose of 50 mg/kgBB/day for the treatment of metastasis in colorectal cancer [23]. The histopathological analysis using H&E staining demonstrated a significant reduction in metastatic nodules after 14 days of treatment, indicating a therapeutic effect. Interestingly, the PECE formulation showed greater efficacy compared to pure Amg. This is parallel with the finding in this study, whereby the formulation of *Amg* in the form of nanoparticles has managed to improve the therapeutic efficacy of this compound.

The percentage of weight loss was calculated for each treatment group during the observation period. In the negative control group, weight loss continued to increase until 14 days, with a percentage of −14.25%. However, the pure *Amg* group showed more controlled weight loss throughout the 14-day therapy, with a percentage of −4.87%. Interestingly, NANO-AMCAL 20 mg showed an increase in body weight, with a percentage increase of 0.24% until day 14, which was not significantly different from the positive control tamoxifen group (2.05%). According to the American Cancer Society (2018), drastic weight loss can be the first visible symptom of several types of cancer, including breast cancer, which triggers an increase in inflammation in the body [56].

In response to tumors that produce pro-inflammatory cytokines, the body’s immune system can trigger an inflammatory response, leading to metabolic changes and hormonal disruptions that affect appetite. This reaction can result in the breakdown of fat and muscle tissue, allowing growing tumors to utilize a large amount of the body’s energy and increase resting energy expenditure (REE), which is the amount of energy burned while at rest. Based on the observed percentage of rat body weight loss, a dose of 20 mg is considered the optimal dose for controlling body weight in test animals with tumors [41]. Additionally, the percentage decrease in average tumor volume was calculated for each treatment group during the observation period. In the negative control group, tumor volume continued to increase by 52.46% until day 14, while pure *Amg* was able to inhibit tumor growth by 5.61%. The NANO-AMCAL 20 mg demonstrated a reduction in tumor volume −17.43% until day 14, which was not significantly different from the positive control tamoxifen group, with a reduction percentage of −20.28%.

Controlling body weight has been found to be able to inhibit the increase in tumor volume. Previous studies have also shown that *Amg* at a dose of 20 mg/kg/day successfully inhibited tumor growth in a mouse model of mammary carcinoma [42]. Hence, based on the percentage reduction in tumor volume observed in the test animals, it can be concluded that a dose of 20 mg is the optimal dose for anti-tumor activity. NANO-AMCAL, which contains 0.33 mg of *Amg*, has been found to be three times more effective at healing breast cancer in Wistar rats than pure *Amg* at a similar quantity (20 mg).

The histopathological analysis showed that negative control breast cancer tissue had poor differentiation and a high grade and was categorized as stage IV metastatic cancer. In comparison, the control rat group showed significant differences. Stage III cancer, which had spread to the lungs, exhibited metastases, and significant differences were observed in the lung tissue of the rats in this group, which were assessed based on Bloom–Richardson criteria. This included features of mitosis, nuclear pleomorphism, and tubule and gland formation (see Figure 15). Meanwhile, in the case of NANO-AMCAL 20 mg, tissue repair increased two-fold compared to pure *Amg*, and it was classified as Stage 1 and equivalent to tamoxifen. More than 75% of tumor cells were arranged in tubules, the number of mitoses was <10, and uniform cell nuclei were observed, which were relatively small with scattered chromatin patterns and the presence of tissue repair patterns. The efficacy of Amg nanoparticles as an anticancer agent depends on the dose, with higher doses showing a better healing effect [28]. Therefore, based on these findings, it can be concluded that NANO-AMCAL 20 mg is the most effective dose against breast cancer cells.

## 4. Conclusions

Based on the characterization test of NANO-AMCAL-loaded Amg, the best variation was alginate with a concentration of 10%, a particle size of 341 nm, and a potential zeta value of +42.1 mV. The solubility and effectiveness of NANO-AMCAL are improved in comparison to the pure Amg in MCF7 breast cancer cell cytotoxicity with an IC_50_ value of 2.744 µg/mL. The NANO-AMCAL with 0.33 mg/g of *Amg* has three times more activity than 20 mg pure *Amg* in suppressing tumor development. This study also evaluated the impact of the different treatments on the rats’ body weight, which can affect cancer progression and related treatments. The group treated with NANO-AMCAL 20 mg and the positive control tamoxifen showed an increase in body weight, which was not observed in the negative control and pure Amg groups. Overall, this study highlights the potential of NANO-AMCAL as an effective treatment option for breast cancer, with superior results compared to pure Amg.

## Figures and Tables

**Table 1:** Formula of NANO-AMCAL.

Ingredients	Amount (grams)	Solvent	Volume (mL)
*Amg*	0.03	Ethanol 96%	10
Chitosan	0.3	Acetic acid 1%	100
TPP	0.06	Distilled water	20
Alginate	0.01	Aquadest^®^ 700C	10

**Table 2:** Grouping of the test animals.

Group	Number (rat)	Treatment	Induction	Therapy
I	4	Normal	-	-
II	4	Control	v	-
III	4	Tamoxifen	v	v
IV	4	NPαM5 mg	v	v
V	4	NPαM10 mg	v	v
VI	4	NPαM20 mg	v	v
VII	4	PαM	v	v

* NPαM = NANO-AMCAL.

**Table 3:** Entrapment efficiency and loading capacity.

Drug Delivery System	Entrapment Efficiency (%)	Loading Capacity (%)
NANO-AMCAL 10%	93.94	5
NANO-AMCAL 25%	89.03	4.8
NANO-AMCAL 50%	85.23	4.4

**Table 4:** Acquired IC_50_ values of active substances and nanoparticle formulations.

Sample Test	IC_50_ Value (µg/mL)
*Amg* Pure	8.350
Cs/TPP/Alg 10%	364.800
Cs/TPP/Amg	6.590
NANO-AMCAL 10%	2.744

**Table 5:** Treatment dosage.

Code	Prescription Name	Quantity	Solvent	Volume
-	Tamoxifen	0.36 mg	NaCMC 0.5%	1 mL/P.O
P.αM	P. *Amg*	20 mg	NaCMC 0.5%	
NPαM	NANO-AMCAL 5 mg	5 mg	Aquadest	
	NANO-AMCAL 10 mg	10 mg	Aquadest	
	NANO-AMCAL 20 mg	20 mg	Aquadest	

## Data Availability

Not applicable.
